# Immunomodulation of Antigen Presenting Cells Promotes Natural Regulatory T Cells That Prevent Autoimmune Diabetes in NOD Mice

**DOI:** 10.1371/journal.pone.0031153

**Published:** 2012-02-15

**Authors:** Martin J. Richer, Danielle J. Lavallée, Iryna Shanina, Marc S. Horwitz

**Affiliations:** Department of Microbiology and Immunology, The University of British Columbia, Vancouver, British Columbia, Canada; Tulane University, United States of America

## Abstract

Progression towards type 1 diabetes (T1D) in susceptible patients is linked to a progressive decline in the capacity of regulatory T cells (Treg) to maintain tolerance. As such, therapies aimed at redressing the failing Treg compartment have been the subject of intense investigation. Treg dysfunction in T1D has recently been linked to a reduced capacity of antigen presenting cells (APCs) to maintain Treg function rather than Treg intrinsic defects. This suggests that therapies aimed simply at addressing the failing Treg compartment are unlikely to provide long-term protection. Here, we demonstrate that modulation of the inflammatory status of CD11b+CD11c− APCs favors the upregulation of protective Tregs in a mouse model of T1D. We further demonstrate that reduced expression of the costimulatory molecule CD40 plays a role in this increased immunoregulatory capacity. Strikingly, Treg upregulation resulted exclusively from an increase in natural Tregs rather than the peripheral conversion of conventional T cells. This suggests that modulation of CD11b+ CD11c− APCs inflammatory properties favors the establishment of natural Treg responses that, unlike adaptive Treg responses, are likely to maintain tolerance to a broad range of antigens. As such, modulation of this APC subset represents a potential therapeutic avenue to reestablish peripheral tolerance and protect from autoimmune diseases such as T1D.

## Introduction

Type 1 diabetes (T1D) is a T-cell mediated autoimmune disease that results from the destruction of the insulin producing β cells of the pancreas. In both humans and non-obese diabetic (NOD) mice, a mouse model of T1D, disease development is progressive and partial peripheral tolerance to islet antigens is only transiently maintained [1]. Several reports have demonstrated an important role for regulatory T cells (Tregs) in the maintenance of peripheral tolerance towards pancreatic self-antigens [2]–[7]. Furthermore, in both mice and humans a progressive loss of Treg suppressive capacities correlates with disease development [8]–[11]. Recent data suggest that the loss of suppressive capacity is not due to Treg intrinsic defects but rather to a decreased capacity of antigen presenting cells (APCs) to maintain Treg function [12], [13]. In NOD mice, CD11b+CD11c− APCs are in part responsible for the decrease in Treg suppressive function and this defect maps to the well-described autoimmune diabetes susceptibility loci Idd3 [12]. Importantly, APCs from T1D patients are similarly defective in their capacity to maintain Treg function [14]. Furthermore, these same APCs present with an inflammatory phenotype that could favor the development of potentially pathogenic IL-17-producing T cells (Th17) [15]. Taken together, this suggests that therapies aimed at modifying the APC response may allow for the reestablishment of peripheral tolerance and allow for long-term protection from T1D. To this effect, it has previously been demonstrated that treatment with glatiramer acetate prevents the induction of experimental autoimmune encephalomyelitis (EAE) in mice by inducing “semi-mature” type II monocytes (M2) that in turn induce protective Tregs [16].

Susceptibility to T1D is dictated by a complex interplay between genetic determinants and environmental influences [17], [18]. Among these environmental factors, viral infections have long been associated with the development of T1D [19]. Particularly, coxsackieviral infections have been described as a common precursor to T1D in patients [19] and the diabetogenic properties of coxsackievirus B4 (CB4) infections have been well documented in mouse models [20]–[22]. We have previously demonstrated that CB4 infection of NOD mice increases lymphocyte infiltration of the pancreas resulting in accelerated disease onset [23]. Furthermore, the pancreatic islet pathology observed following CB4 infection is characteristic of the pathology associated with spontaneous disease progression in NOD mice. As such, autoimmune diabetes acceleration following CB4 infection represents an experimentally-controlled model that allows for rapid modeling of therapies aimed at modulating diabetes onset.

Our prior work demonstrated that one consequence of CB4 infection was the infection of pancreatic β cells. Subsequently, antigen presenting cells (APCs) engulfed the infected β cells for T cell recognition and stimulation leading to an autoimmune attack upon the insulin producing islet cells. Transfer of APCs from infected NOD mice induced disease in non-diabetic recipient mice [24], [25]. Later, we observed that TGF-β through transgenic expression or systemic administration established protection from CB4-induced autoimmune diabetes [23]. This protection correlated with the presence of “semi-mature” CD11b+CD11c− APCs expressing lower surface levels of costimulatory molecules as well as an increase in pancreatic Tregs [23]. Interestingly, the capacity of the host to clear viral infection remained unaffected. This strongly suggested that APCs could be manipulated in order to prevent autoimmunity without affecting the host's response to infection. Herein, we demonstrate that a reduction in the inflammatory properties of CD11b+CD11c− APCs is sufficient to increase levels of pancreatic Tregs. Further, this increase in Tregs which is observed following infection in the localized presence of TGF-β is the result of an increase in the numbers of natural Tregs rather than the phenotypic conversion of conventional T cells to a Treg cells. Our results suggest that the development of therapeutics aimed at modulating APC function and increasing the levels of natural Tregs may represent a powerful tool for reestablishing peripheral tolerance to beta cell antigen and providing long-term protection from T1D.

## Results

### CD11b+CD11c− APCs demonstrate reduced inflammatory properties following CB4 infection in the context of TGF-β

**Figure 1:**
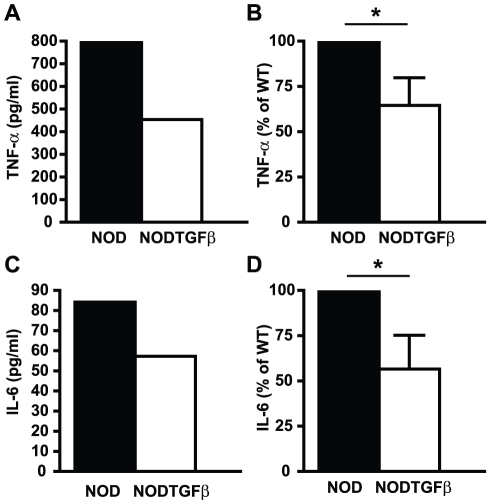
CD11b+CD11c− APCs produce lower levels of inflammatory cytokines following CB4 infection in the context of TGF-β. *Ex vivo* production of A, B) TNF-α or C,D) IL-6 from CD11b+CD11c− APCs FACS sorted from CB4 infected NOD (black bar) or NODTGFβ (white bar) mice. Cytokine levels were measured from culture supernatants following 24 hours of incubation. Panels A,C are representative data from one experiment with pooled mice of each genotype while panels B,D represent mean + s.e.m of cytokine levels from 6 separate experiments normalized to the cytokine levels produced by WT NOD mice infected with CB4 in each separate experiments.

We previously demonstrated that CB4 infection in the context of TGF-β protects from the induction of autoimmune diabetes [23]. Protection was mediated by increased Treg cell numbers in the pancreas and correlated with the reduced maturation of CD11b+CD11c− APCs as measured by costimulatory molecule expression [23]. Here, we asked whether infection in the presence of TGF-β affected other inflammatory properties of these APCs. CD11b+CD11c− (a population containing predominantly monocytes and macrophages) and CD11c+ (predominantly dendritic cells) cells from NOD and NODTGFβ mice were sorted by flow cytometry at day 7 following CB4 infection and cultured *ex vivo* for 24 hours. Cytokine secretion in the culture supernatant was measured. Relative production of the inflammatory cytokines TNF-α (Figure 1A,B) and IL-6 (Figure 1C,D) from CD11b+CD11c− APCs purified from CB4 infected NODTGFβ mice was significantly reduced compared to the same population purified from CB4 infected NOD mice. No significant production of IL-12p70, IFN-γ or IL-2 was observed (data not shown). Furthermore, while we observed no significant production of the anti-inflammatory cytokine IL-10 (data not shown), we observed a trend for increased TGF-β production from CD11b+CD11c− APCs purified from infected NODTGFβ mice although this difference did not reach statistical significance (Figure S1). This suggests that the primary effect of TGF-β in this model is to reduce the production of inflammatory cytokines by APCs rather than to directly increase their production of anti-inflammatory cytokines. Interestingly, infection in the context of TGF-β did not affect the capacity of DCs to produce inflammatory cytokines (Figure S2). This is supported by our previously published data demonstrating that infection in the context of TGF-β did not affect the maturation status of DCs [23]. Taken together, these data demonstrate that CD11b+CD11c− APCs with reduced inflammatory properties are generated following CB4 infection in the context of TGF-β.

**Figure 2:**
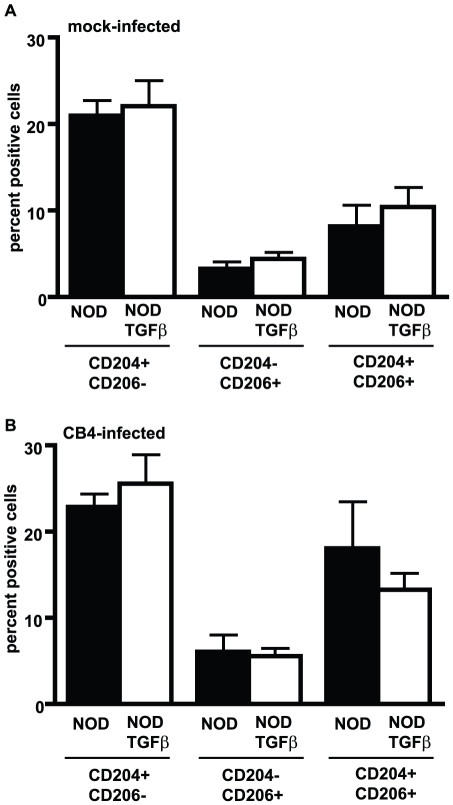
CB4 infection does not increase expression of type II monocyte/macrophages markers on CD11b+CD11c-APCs. A) Percentage of CD11b+CD11c− APCs from mock-infected NOD (black bars) or NODTGFβ (white bars) mice positive for surface expression of CD204, CD206 or CD204 and CD206. B) Percentage of CD11b+CD11c− APCs from NOD (black bars) or NODTGFβ (white bars) mice positive for surface expression of CD204, CD206 or CD204 and CD206 7 days following CB4 infection. Data represent mean + s.e.m from 3 separate experiments, n = 9 per group.

Reduced production of inflammatory cytokines has been previously associated with alternatively activated or type II monocytes/macrophages (M2) [16]. As such, we asked whether infection in the context of TGF-β resulted in the polarization of monocytes/macrophages towards an M2 phenotype. To address this question, we measured surface expression of the macrophage scavenger receptor (MSR-A, CD204) and of the macrophage mannose receptor (MMR, CD206), two markers associated with M2 monocytes/macrophages [26], and of the negative costimulatory molecule programmed death ligand-1 (PD-L1, B7-H1), a molecule associated with the maintenance of peripheral tolerance [27]. No significant differences were observed in the percentage of CD11b+CD11c− APCs expressing CD204 or CD206 only or both CD204 and CD206 between NOD and NODTGFβ mice prior to infection (Figure 2A) or at day 7 PI (Figure 2A, B). Similarly, we observed no significant differences in PD-L1 expression between CD11b+CD11c− APCs from NOD or NODTGFβ mice at day 7 following CB4 infection (Figure S3). This suggests that following CB4 infection in the context of TGF-β, monocytes/macrophages with a typical M2 phenotype are not likely generated.

### CD11b+CD11c− APCs with reduced inflammatory properties are sufficient to increase Treg levels in uninfected NOD mice

**Figure 3:**
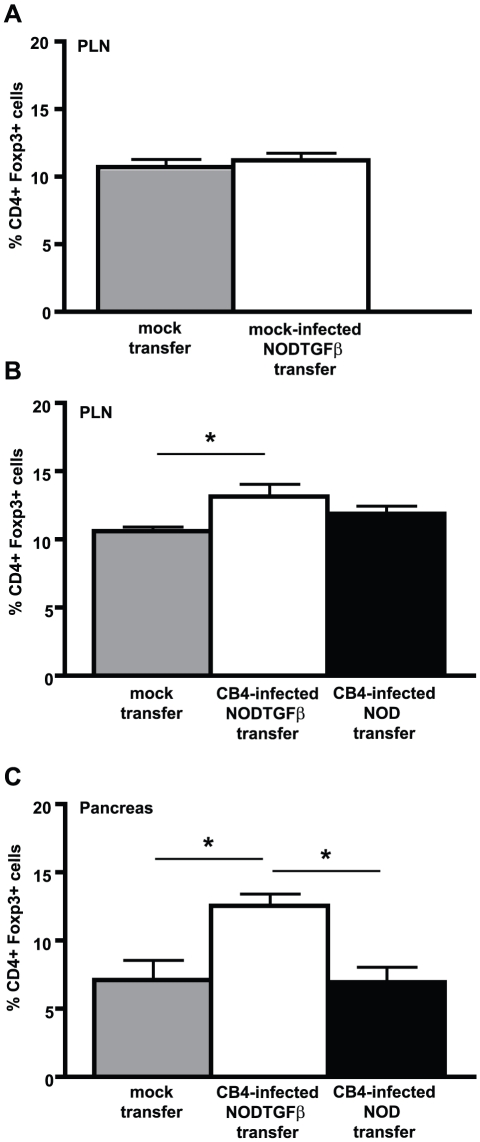
Adoptive transfer of CD11b+CD11c− APCs from CB4-infected NODTGFβ mice is sufficient to increase Treg levels in uninfected NOD mice. Average percentage of CD4+ T cells expressing Foxp3 in A, B) the PLNs or C) the pancreas of 10–12 week uninfected NOD recipients mock-transferred with DMEM (grey bars) or adoptively transferred with CD11b+CD11c− APCs FACS sorted from A) mock-infected NODTGFβ mice (white bars) or B, C) CB4 infected NODTGFβ (white bars) or NOD mice (black bars). Data represent mean + s.e.m. from at least 3 separate experiments (n = at least 6 mice per group).

Protection from autoimmune diabetes in mice expressing TGF-β is mediated by an increased pancreatic Treg presence and correlated with the induction of a “semi-mature” population of CD11b+CD11c− APCs [23]. As “semi-mature” APCs have been previously associated with Treg induction [16], we asked whether CD11b+CD11c− APCs were responsible for the increase of protective Tregs observed in NODTGFβ mice following CB4 infection. CD11b+CD11c− APCs were sorted by flow cytometry from NOD or NODTGFβ mice from mock-infected mice or at day 7 PI with CB4 and adoptively transferred to uninfected 10–12 week old NOD mice. While transfer of CD11b+CD11c− APCs from uninfected NODTGFβ mice was not sufficient to increase Treg levels (Figure 3A), we observed a significant increase in Treg levels in the PLNs (Figure 3B) and pancreas (Figure 3C) of NOD mice that had been adoptively transferred with CD11b+CD11c− APCs from NODTGFβ mice compared to mock-transferred NOD recipients. Conversely, adoptive transfer of the same population purified from infected WT NOD mice was not sufficient to significantly increase Treg levels in either the PLNs (Figure 3B) or the pancreas (Figure 3C). This data builds on our previous observations [23] and further strengthens the hypothesis that CD11b+CD11c− APCs are central to the induction of Tregs that functionally suppress the induction of autoimmune diabetes following CB4 infection in the context of TGF-β. Importantly, transfer of CD11b+CD11c− APCs from uninfected NODTGFβ mice (Figure 3A) or NODTGFβ mice infected with CB3, a closely related virus strain that unlike CB4 does not infect pancreatic beta cells, was not sufficient to increase Treg levels (Figure S4). This suggests that infection of pancreatic beta cells and subsequent presentation of self-antigen by “semi-mature” CD11b+CD11c− APCs is an essential requirement for the induction of Tregs *in vivo*.

### Reduced expression of CD40 on CD11b+CD11c− APCs favors Treg upregulation

**Figure 4:**
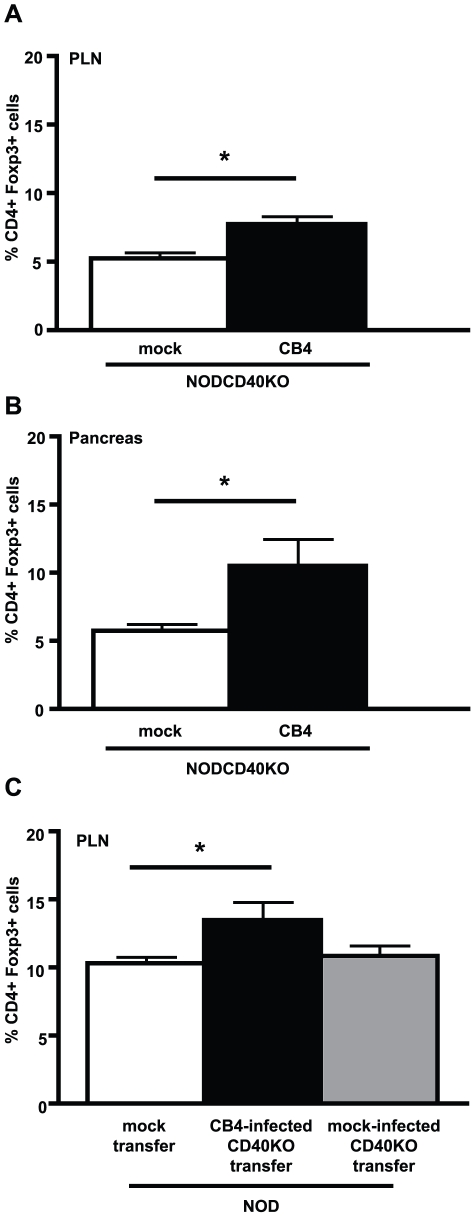
Absence of CD40 on CD11b+CD11c− APCs is sufficient to increase Treg levels following CB4 infection. Average percentage of CD4 T cells expressing Foxp3 in the A) PLNs or B) pancreas of NODCD40KO mice mock-infected with DMEM (white bars) or infected with CB4 (black bars) (n = at least 10 per group). C) Average percentage of CD4+ T cells expressing Foxp3 in the PLNs of uninfected 10–12 week old NOD mice mock-transferred with DMEM (white bar) or adoptively transferred with CD11b+CD11c− FACS sorted from CB4 infected NODCD40KO (black bar) or mock-infected NODCD40KO mice (grey bars). Data represent mean + s.e.m from at least 3 separate experiments (n = at least 7 per group).

CD11b+CD11c− APCs from NODTGFβ mice have previously been demonstrated to express lower surface levels of costimulatory molecules following CB4 infection [23]. As such, we asked whether this “semi-mature” phenotype was linked to their capacity to induce Tregs. To address the role of CD40, NOD mice deficient for CD40 (NODCD40KO) were infected with CB4. As these mice lack transgene-driven pancreatic expression of TGF-β, this model allow us to directly test the hypothesis that the reduction of CD40 on the surface of CD11b+CD11c− APCs following infection in the context of TGF-β is part of the mechanism that favors Treg increases. At day 7 PI, we observed a significant increase in Treg levels in the PLNs (Figure 4A) and pancreas (Figure 4B) of infected NODCD40KO mice compared to mock-infected mice suggesting an important role for reduced surface expression of CD40 in conferring CD11b+CD11c− APCs with the capacity to induce Tregs. In order to confirm the contribution of CD11b+CD11c− APCs in increasing the Treg levels in NODCD40KO mice following CB4 infection, CD11b+CD11c− APCs were sorted by flow cytometry from CB4 infected NODCD40KO mice and adoptively transferred to uninfected 10–12 week old NOD mice. As opposed to what we observed following CD11b+CD11c− APC transfer from CB4 infected CD40-competent NOD mice (Figure 3), adoptive transfer of CD11b+CD11c− APCs from CB4 infected CD40-deficient NOD mice, but not mock-infected CD40-deficient NOD mice, significantly increased levels of Tregs in the PLNs compared to mock-transferred mice (Figure 4C). Taken together, these data suggest that the protection from autoimmune diabetes conferred by pancreatic expression of TGF-β is in part dictated by the reduction of CD40 expression on CD11b+CD11c− APCs that, in turn, favors increases in Treg levels.

### Treg upregulation results from an increase in natural Tregs rather than conversion of conventional T cells to a Treg phenotype

**Figure 5:**
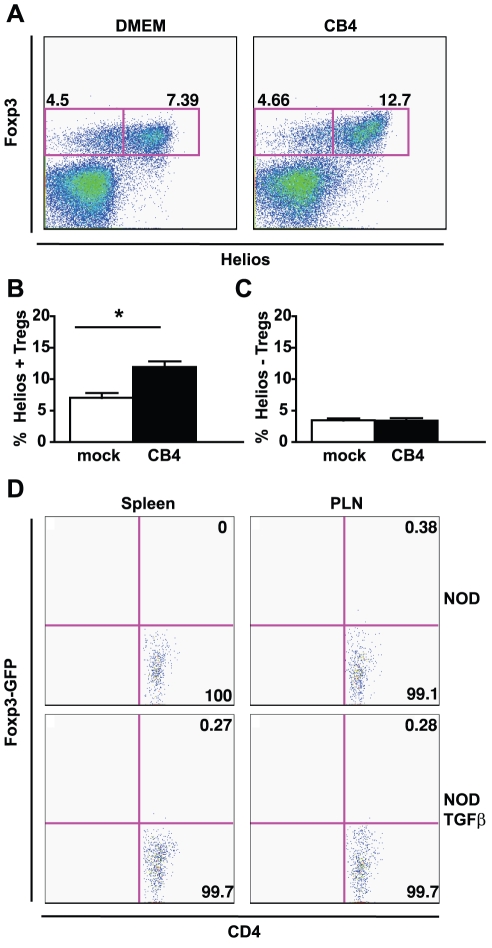
CB4 infection in the context of TGF-β does not induce conversion of naïve T cells to adaptive Tregs but rather increases the levels of natural Tregs. A) Representative flow cytometry plots of Foxp3 and Helios expression by CD4+ T cells from the PLNs of mock-infected (left panel) or CB4-infected NODTGFβ mice at day 7PI. B) Average percentage of Helios+ cells among CD4+Foxp3+ Tregs from the PLNs of NODTGFβ mice with increased Treg levels at day 7 PI with CB4 (black bars) or mock-infection with DMEM (white bars). C) Average percentage of Helios- cells among CD4+Foxp3+ Tregs from the PLNs of NODTGFβ mice with increased Treg levels at day 7 PI with CB4 (black bars) or mock-infection with DMEM (white bars). Data represent mean + s.e.m from at least 2 separate experiments (n = at least 4 per group). D) Representative flow cytometry plots of CD4 and GFP (Foxp3) expression of donor (Thy1.1+) cells recovered from the spleen (left panels) or PLNs (right panels) of NOD (top panels) or NODTGF-β (bottom panels) recipient mice at day 7 following infection with CB4. Data is representative of 2 separate experiments, n = 3 for NOD recipients and n = 6 for NODTGFβ recipients.

To better understand the mechanism of protection from T1D, experiments were designed to determine how Tregs levels are increased following CB4 infection in the local presence of TGF-β. It has been well documented that T cell stimulation in the presence of TGF-β can convert naïve T cells to a Treg phenotype by inducing Foxp3 expression [28]–[31]. As such, we asked whether Tregs increases observed in NODTGFβ mice following CB4 infection resulted from phenotypic conversion of conventional effector T cells to regulatory T cells or rather an increase in the numbers of natural regulatory T cells. To differentiate increases in natural Tregs from the induction of adaptive Tregs, we measured the percentage of Tregs expressing the transcription factor Helios, a recently described marker specifically expressed by thymically-derived natural Tregs but not by Tregs induced by peripheral conversion [32]. Following infection in the localized presence of TGF-β, we observed a significant increase in the percentage of Tregs that express Helios in the PLN of mice with increased Treg levels (Figure 5 A,B). Conversely, the percentage of Tregs not expressing Helios remained unchanged following infection. These results strongly suggest that the increased Treg levels observed following infection in the localized presence of TGF-β result from an increase in natural Tregs rather than the conversion of conventional T cells.

To confirm the observed lack of T cell conversion to Tregs, experiments were designed to test whether CB4 in the context of TGF-β was sufficient to induce conversion of conventional T cells to a regulatory phenotype by inducing the expression of foxp3. NODThy1.1 mice were crossed with previously described NOD mice expressing a Foxp3^GFP^ reporter [33], [34]. Conventional T cells were sorted by flow cytometry as CD4+GFP− cells and adoptively transferred in 10–12 week old NOD or NODTGFβ recipients (expressing the Thy1.2 allele allowing for the differentiation of donor cells). Mice were challenged with CB4 at 24 hours post-transfer and analyzed by flow cytometry at day 7 PI. These experiments were performed using viral infection rather than adoptive transfer as viral infection yields a more robust increase in Treg levels allowing for a better characterization of the induced population. CD4 T cells of donor origin (Thy1.1+) were readily identifiable in the spleen, PLNs and pancreas of both NOD and NODTGFβ mice (Figure 5 and data not shown). However, none of the recovered donor CD4 T cells were positive for GFP in either NOD or NODTGFβ recipients indicating that infection in the local presence of TGF-β does not induce Foxp3 expression in CD4 T cells (Figure 5C). These results confirm the data obtained by helios staining and demonstrate that increases in Tregs observed in this model result from an increase in natural regulatory T cells.

## Discussion

It is becoming increasingly clear that defects in peripheral tolerance are linked to the development of T1D. Specifically, a decline in the capacity of Tregs to suppress autoreactive T cells has been associated with disease progression in both humans and mice [35]. Recent evidence has demonstrated that this decline in suppressive capacity is not due to Treg intrinsic defects but rather to changes at the level of APCs [12]–[14]. Here, we demonstrate that CD11b+CD11c− APCs with reduced inflammatory properties are accountable for an increase in pancreatic Treg levels.

A link between the CD11b+CD11c− subset of APCs and the declining function of Tregs in NOD mice has been recently established [12]. As such, our findings suggest that therapies aimed at targeting the inflammatory properties of CD11b+CD11c− APCs may represent a long-term solution to reestablishing peripheral tolerance to pancreatic antigens. In this regard, it has been demonstrated that glatiramer acetate treatment protects from autoimmunity in a mouse model of multiple sclerosis (MS) by acting at the levels of APCs. Specifically, drug treatment reduced the maturation status and inflammatory properties of CD11b+ monocytes and increased their capacity to induce Tregs [16]. The similarities in phenotypes observed in the MS model and in NOD mice infected with CB4 in the context of TGF-β suggest that glatiramer acetate treatment may represent a potential therapeutic avenue for the treatment of T1D. Interestingly, glatiramer acetate treatment of NOD mice increased Treg levels and partially protected from cyclophosphamide-induced T1D acceleration [36]. Similarly, treatment with complete Freund's adjuvant has also been demonstrated to modulate the capacity of APCs from the NOD mice to maintain regulatory T cell function and protect from autoimmune diabetes [37]. In addition, recent studies have demonstrated that recombinant adeno-associated virus can be used to express constructs under the control of an insulin promoter specifically within the islets [38], [39]. This suggests that this approach could be adopted to modulate the inflammatory properties of APCs directly within the pancreas and these studies are currently ongoing with Kieffer and colleagues. Taken together, these data strongly suggest that CD11b+CD11c−APCs represent a key target for therapies aiming to reestablish tolerance and protect from T1D. Importantly, our prior work clearly established that manipulating the inflammatory status of these APCs does not affect the capacity of the host to respond to viral infection strongly suggesting that targeting this subset of APCs would not result in generalized immunosuppression [23].

The capacity of CD11b+CD11c− APCs to increase Tregs following infection in the localized presence of TGF-β is correlated with three important features. First, we previously demonstrated that CD11b+CD11c− APCs present with a “semi-mature” phenotype characterized by reduced surface expression of costimulatory molecules [23]. Here, we further demonstrate that reduced expression of CD40 plays a role in the heightened capacity of CD11b+CD11c− APCs from NOD mice to increase Treg levels. This strongly suggests that while CD40 signals are likely involved in the acceleration of T1D by CD11b+CD11c− APCs following CB4 infection, reduction or absence of these signals tips the balance towards Treg induction. This is supported by data demonstrating that blockade of CD40/CD154 interactions through genetic ablation of either molecule or blocking antibody treatment is associated with decreased alloimmune responses and increased transplant tolerance [40]. Interestingly, CD40 deficiency has previously been demonstrated to result in reduced Treg frequency in the periphery suggesting a role for CD40 in Treg homeostasis. While we observed that, similar to other mouse models [41], [42], baseline Treg levels were lower in NOD mice lacking CD40 compared to WT NOD, our results clearly demonstrate that under inflammatory conditions reduced CD40 expression favors Treg increases. This suggests that under inflammatory conditions, CD40 expression may act to control the balance between activation of effectors and immunoregulation by Tregs [7], [43]. As such, therapies aimed at reducing CD40 expression on CD11b+CD11c− may prove particularly effective at reestablishing Treg function and peripheral tolerance. Second, CD11b+CD11c− APCs generated following CB4 infection produce lower levels of the inflammatory cytokines TNF-α and IL-6. This may have several effects on disease progression. In particular, autoimmune diabetes acceleration following CB4 infection has been hypothesized to progress through a bystander activation mechanism that relies upon presentation of previously sequestered antigens in an inflammatory milieu [20]. As such, reduced inflammation likely curtails the activation and/or expansion of autoreactive T cells and this will be the subject of future investigation. IL-6 has previously been associated with blocking Treg suppressive capacities [44] and preventing the expression of the Treg specific transcription factor Foxp3 in order to favor the generation of Th17 clones [45]. As such, decreased IL-6 production may be linked to the capacity of CD11b+CD11c− APCs from NODTGFβ mice to increase Treg levels. Third, infection of pancreatic beta cells appears to be a critical requirement for the induction of Tregs by CD11b+CD11c− APCs. This strongly suggests that the presentation of pancreatic self-antigens by semi-mature APCs favors the generation of protective Tregs in this model. This likely occurs directly within the pancreas, however, attempts to determine this histologically have been hampered by the destruction of exocrine tissue induced by coxsackieviral infection. Furthermore, depletion experiments aiming to confirm the data from the adoptive transfer experiments presented in this manuscript are hampered by the critical role of macrophages in the response to viral infection [46]. As such, any attempt to deplete macrophages in this system would result in virus-induced mortality.

Taken together, our data demonstrate that protection from T1D is linked to a decrease in the capacity of CD11b+CD11c− APCs to activate autoreactive responses and an increase in their immunoregulatory properties. As such, therapies aimed at this particular APC subset may have a two-fold protective effect by reducing the capacity of these cells to drive autoimmune pathology and favoring their capacity to induce protective mechanisms. Interestingly, despite the localized expression of TGF-β directly within the pancreatic islets, we observed that APCs isolated from the spleen of CB4-infected NODTGFβ mice present with immunoregulatory properties. This suggests that while APCs appear to acquire antigen and become tolerogenic directly within the pancreas they retain the ability to recirculate to mediate their immunomodulatory functions. This is supported by data demonstrating that NODTGFβ mice are protected for coxsackievirus-induced autoimmune myocarditis and that this protection is dependent upon infection of the pancreas and correlates with modulation of APCs [47], [48]. This strongly suggests that locally modulated APC population can induce protection from autoimmunity at distal sites.

The generation of protective Tregs following infection in the context of TGF-β may represent an extension of a natural process that allows maintenance of tolerance to self-antigens, following viral infections. Infections with LCMV have long been described to protect NOD mice from the development of T1D [49]. This protection is associated with increases in several immunoregulatory mechanisms that are likely activated to prevent immunopathology following the resolution of an antiviral response. In particular, this was associated with increased TGF-β production and increased levels of Tregs that protect from the induction of autoimmune diabetes [50]. This suggests that any mechanism that serves to increase the production of TGF-β may be sufficient to reestablish long-term tolerance and protect from T1D.

Finally our data demonstrate that the heightened Treg levels observed following infection in the context of TGF-β are not due to conversion of naïve T cells to a regulatory phenotype. Rather, our data demonstrate that the increase in Tregs results from an increase in Helios expressing natural Tregs [32]. This suggests than in addition to its well-described capacity to contribute to the conversion of conventional T cells to a Treg phenotype, stimulation of T cells in the presence of TGF-β can also result in an overall increase in the presence of natural Tregs. Although the mechanism responsible for this increase in natural Tregs remains undefined, our preliminary data suggest that this does not result from an increase in Treg survival or an increase in the proliferative capacity of Tregs (Richer and Horwitz, unpublished observations). Of particular interest, Foxp3 expression was recently described to be unstable in NOD mice and loss of Foxp3 expression yields inflammatory cells that can contribute to T1D pathogenesis [51]. It is possible that CB4 infection in the context of TGF-β may function to stabilize Foxp3 expression and this warrants further investigation. Interestingly, Thornton and colleagues have recently demonstrated that natural Tregs expressing Helios appear less likely to gain an inflammatory phenotype than peripherally converted Tregs lacking Helios [32]. As such, therapies aimed at increasing the levels of Helios expressing natural Tregs may prove particularly effective at preventing autoimmune diseases such as T1D. Furthermore, the relative contribution of natural and adaptive Tregs in the progression of autoimmune diabetes in NOD mice remains to be addressed and should be the subject of further investigation.

In conclusion, our results demonstrate that the inflammatory properties of CD11b+CD11c−APCs can be modulated to yield a subset of APCs with immunoregulatory properties. We provide evidence that reducing the inflammatory properties of this APC subset is sufficient to switch the effects of CB4 infection from a diabetogenic [24] to a protective role. Taken together with evidence that the decline in Treg function associated with disease progression is not due to Treg intrinsic defects but rather to a reduction in the capacity of this APC subset to maintain Treg function [12], this argues for the targeting of this APC subset as a powerful therapeutic tool for the long-term reestablishment of peripheral tolerance to beta cell antigens.

## Materials and Methods

### Mice

NOD/ShiLtJ mice were obtained from The Jackson Laboratory (Bar Harbor, USA). NODTGFβ transgenic mice expressing TGF-β under the control of the human insulin promoter were generated in the laboratory of Dr. N. Sarvetnick (University of Nebraska Medical Center, Omaha, NE, USA) and have been previously characterized [23], [52]. NODCD40KO and NODThy1.1 and NODFoxp3^GFP^ mice were obtained from the JDRF Center on Immunological Tolerance at Harvard Medical School. All mice were bred and maintained in our rodent facility and tested for diabetes prior to infection. Mice with two consecutive non-fasting blood glucose readings of >300mg/dl were considered diabetic. Animals experiments were approved, conform and were performed to the guidelines of the Animal Care Committee at the University of British Columbia which ensures that appropriate care, including adequate veterinary care, is given to animals in all stages of life and in all experimental situations in compliance with Canadian Council on Animal Care (“CCAC”) guidelines and CALAM Standards of Veterinary Care. This work was approved by the UBC ACC and the approved animal protocol number is AO8-0622.

### Virus

Stocks of CB4 Edwards strain 2 and CB3 Nancy strain were obtained and prepared as described previously [47], [53]. 10–12 week old mice were infected intraperitoneally with sublethal doses of 400 plaque forming units (PFU) of virus diluted in DMEM, mock-infected mice received an equivalent volume of DMEM.

### Flow cytometry

Single cell suspensions were stained for the appropriate markers and analyzed by flow cytometry on an LSRII cell analyzer (BD Biosciences, Missisauga, Canada). Fluorescently conjugated antibodies directed against CD11b (clone M1/70), CD11c (clone N418), PD-L1 (clone M1H5), CD4 (clone L3T4), CD25 (clone PC61), BCL2 (clone 10C4) and Foxp3 (clone FJK-16s) were purchased from eBiosciences (San Diego, USA). Fluorescently conjugated antibodies directed against CD204 (clone 2F8) and biotin-conjugated antibodies directed against CD206 (clone MR5D3) were purchased from AbD Serotec (NC, USA). Fluorescently conjugated antibodies directed against Helios were purchased from BioLegend (San Diego, USA). Data were analyzed with FlowJo software (Tree Star).

### APC adoptive transfer

Single cell suspensions were generated from spleens of CB4 infected mice at day 7 post-infection (PI). Cells were stained with fluorescently conjugated antibodies directed against CD11b and CD11c and sorted on a FACSAria flow cytometer (BD Bioscience, Missisauga, Canada). 2×10^5^ purified CD11b+CD11c− diluted in DMEM were adoptively transferred intraperitoneally into uninfected NOD mice. Spleens, pancreata and pancreatic lymph nodes (PLNs) were harvested at day 7 post-transfer for Treg analysis. Mock-transferred mice received an equivalent volume of DMEM.

### Ex vivo cytokine analysis

CD11b+ CD11c− cells and CD11c+ cells were sorted by flow cytometry as described above and 1×10^5^ cells were cultured for 24 hours at 37°C in IMDM containing 10% fetal bovine serum. Supernatants were harvested and cytokine levels were measured using a BD CBA inflammation kit (BD Bioscience, Missisauga, Canada) or a Mouse TGF-β1 ELISA Ready-SET-Go! kit from eBioscience (San Diego, USA) Samples were prepared according to manufacturer's instructions and bead array samples were analyzed on a BDFacsArray equipped with FCAP software (BDBiosciences). Cytokine levels were normalized and are presented as a percentage of cytokine production compared to similarly infected WT NOD mice.

### Isolation of pancreatic infiltrating cells

Pancreata were isolated from infected NOD and NODTGFβ mice and mechanically disrupted. Single cell suspensions were treated for 10 minutes at 37°C in a PBS solution containing 1 mg/ml of collagenase. Recovered cells were stained for flow cytometry.

### Treg conversion assay

Single cell suspension from NODFoxp3GFPThy1.1 mice were stained with fluorescently conjugated antibodies directed against CD4 and sorted by flow cytometry. 2×10^6^ CD4+GFP− (non-Tregs) cells were adoptively transferred intraperitoneally into NOD mice or NODTGFβ mice 24 hours prior to infection with 400 pfu of CB4. At day 7 post-infection, conversion to a Treg phenotype was analyzed by measuring GFP (Foxp3) expression in CD4+Thy1.1+ cells from the spleen, pancreas and PLNs.

### Statistical analysis

Statistical analysis was performed with Prism GraphPad software. The unpaired Student's t-test was used for statistical analysis. A P value of less than 0.05 was considered significant.

## Supporting Information

Figure S1 **CD11b+CD11c− APCs do not produce significantly higher levels of TGF-β following CB4 infection in the context of TGF-β.** *Ex vivo* production TGF-β from CD11b+CD11c− APCs FACS sorted from CB4 infected NOD (black bar) or NODTGFβ (white bar) mice. Cytokine levels were measured from culture supernatants following 24 hours of incubation. Data represent mean + s.e.m of normalized cytokine levels from pooled mice in 3 separate experiments.
(EPS)

Figure S2 **CB4 infection in the context of TGF-β does not affect the capacity of DCs to produce cytokines.** *Ex vivo* production of A) TNF-α or B) IL-6 from CD11c+ DCs FACS sorted from CB4 infected NOD (black bar) or NODTGFβ (white bar) mice. Data represent mean + s.e.m of normalized cytokine levels from pooled mice in 3 separate experiments.
(EPS)

Figure S3 **CD11b+CD11c− APCs from NODTGFβ mice do not express increased levels of PD-L1 compared NOD mice following CB4 infection.** A) Representative histogram of surface PD-L1 expression on CD11b+CD11c− APCs from NOD (black line) or NODTGFβ (grey line) mice at day 7 following CB4 infection. B) Average mean fluorescence intensity of PD-L1 expression on the surface of CD11b+CD11c− APCs from NOD (black bar) or NODTGFβ (white bars) mice at day 7 following CB4 infection. Data represent mean + s.e.m. from 2 separate experiments (n = at least 5 per group.).
(EPS)

Figure S4 **Adoptive transfer of CD11b+CD11c− APCs from CB3-infected NODTGFβ mice is not sufficient to increase Treg levels in uninfected NOD mice.** Average percentage of CD4+ T cells expressing Foxp3 in the PLNs of 10–12 week uninfected NOD recipients mock transferred with DMEM (grey bars) or adoptively transferred with CD11b+CD11c− APCs FACS sorted from CB3 infected NODTGFβ (white bars) mice. Data represent mean + s.e.m. from 3 separate experiments (n = at least 6 per group).
(EPS)
